# Public Support for Electronic Cigarette Regulation in Hong Kong: A Population-Based Cross-Sectional Study

**DOI:** 10.3390/ijerph14070709

**Published:** 2017-06-30

**Authors:** Yee Tak Derek Cheung, Man Ping Wang, Sai Yin Ho, Nan Jiang, Antonio Kwong, Vienna Lai, Tai Hing Lam

**Affiliations:** 1 School of Nursing, The University of Hong Kong, Hong Kong, China; mpwang@hku.hk; 2 School of Public Health, The University of Hong Kong, Hong Kong, China; syho@hku.hk (S.Y.H.); hrmrlth@hku.hk (T.H.L.); 3 Department of Population Health, School of Medicine, New York University, New York, NY 10016, USA; Nan.Jiang@nyumc.org; 4 Hong Kong Council on Smoking and Health, Hong Kong, China; iphltd@netvigator.com (A.K.); vlai@cosh.org.hk (V.L.)

**Keywords:** electronic nicotine delivery devices, public opinion, public policy, surveillance and monitoring, advocacy

## Abstract

This study aimed to gauge the Hong Kong’s public support towards new e-cigarette regulation, and examine the associated factors of the support. We conducted a two-stage, randomized cross-sectional telephone-based survey to assess the public support towards the banning of e-cigarette promotion and advertisement, its use in smoke-free venues, the sale to people aged under 18, and regulating the sale of nicotine-free e-cigarettes. Adults (aged 15 years or above) who were never smoking (*n* = 1706), ex-smoking (*n* = 1712) or currently smoking (*n* = 1834) were included. Over half (57.8%) supported all the four regulations. Banning of e-cigarette promotion and advertisement (71.7%) received slightly less support than the other three regulations (banning of e-cigarette use in smoke-free venues (81.5%); banning of e-cigarette sale to minors (93.9%); sale restriction of nicotine-free e-cigarettes (80.9%)). Current smokers, and perceiving e-cigarettes as less harmful than traditional cigarettes or not knowing the harmfulness, were associated with a lower level of support. Our findings showed a strong public support for further regulation of e-cigarettes in Hong Kong. Current stringent measures on tobacco and e-cigarettes, and media reports on the harmfulness of e-cigarettes may underpin the strong support for the regulation.

## 1. Introduction

Electronic nicotine delivery systems, or e-cigarettes, have been advertised as smoking cessation tools in some countries [1,2]. Only a few randomized trials on the effectiveness of e-cigarettes for smoking cessation have been done [3,4], and there has been no evidence that e-cigarettes have greater effectiveness than nicotine replacement therapy [5,6]. Several studies suggested that the effectiveness increased when liquid-refillable e-cigarettes are used [7,8], used daily [7], or used in a less restrictive e-cigarette regulatory environment [9]. Some laboratory studies showed that the aerosol emits lower amounts of harmful substances than traditional cigarettes [10,11]. Based on the existing evidence, e-cigarettes are suggested as cessation aids by some public health experts, especially when the first-line cessation medications fail to increase abstinence [12]. Apart from the potential benefits, e-cigarettes are not totally harmless. E-liquids and aerosols still contain harmful pollutants [13,14,15]. Use of e-cigarettes is associated with increase in airway resistance [13,16], respiratory viral infections [17], and respiratory symptoms [18]. The safety of inhaling the flavor ingredients through e-cigarettes has not been approved [19]. Also, e-cigarettes may serve as a “gateway” to cigarette-smoking in adolescents [20,21].

To balance the risks with the potential benefits from e-cigarettes, various levels of e-cigarette regulation have been implemented worldwide. Less controversial policies include minimum age for e-cigarette purchase and the ban on advertising and promotion, which have been implemented to prevent e-cigarette initiation particularly among youth and non-cigarette smokers. Also, the ban on vaping at public places has been implemented to protect people from being exposed to second-hand e-cigarette aerosol. As of August 2016, twenty-two countries require a minimum legal age for e-cigarette purchase [22]. Forty-eight countries have legislations to restrict e-cigarette advertising or promotion [22]. Thirty-one countries have imposed nation-wide legislation to prohibit e-cigarette use in public spaces [22]. These policies were generally supported by the public [23,24,25,26]. In contrast, restriction of availability is more debatable. Reduction of accessibility and “medicalization” of e-cigarettes (e.g., licensing retailers who sell e-cigarettes) may restrain cigarette smokers to use them as smoking cessation aids, but the permission for selling e-cigarettes as consumer products may re-normalize smoking behavior as using e-cigarettes mimics conventional smoking behavior. Public support in this policy domain varies with countries and smoking status. For instance, licensing e-cigarette retailers are supported by the US general public [26], but smokers in the UK and vapers in Australia tend to oppose policies that reduce the availability and accessibility [27,28]. Currently, 23 countries prohibit all kinds of e-cigarette sale, and 11 countries restrict only the sale of nicotine-containing e-cigarettes [22]. No previous studies have examined the public support for restricting the availability of nicotine-free e-cigarettes. None of the aforementioned studies on public support for e-cigarette regulations were from Asian countries, where e-cigarette marketing is growing.

Hong Kong has no regulation on e-cigarettes specifically, but a few existing tobacco control policies have already covered e-cigarettes. Similar to many countries, any product containing nicotine is regarded as a pharmaceutical product that requires registration before sale and distribution. Thus, e-cigarettes in the local market in Hong Kong are claimed to be nicotine-free, and no nicotine-containing e-cigarettes have been registered for sale as of June 2017 [29]. Marketing of nicotine-free e-cigarette is not restricted in Hong Kong [30]. Nicotine-free e-cigarettes are available in retail stores and, via internet or social media, without age restrictions. Local promotion of nicotine-free e-cigarettes emphasizes their effectiveness for smoking cessation, and their lower harmfulness than traditional cigarettes, which have yet to be confirmed. The grey area of permitting the sale of nicotine-free cigarettes also exists in many other countries such as Canada, Australia, Korea and Japan.

Most Hong Kong residents (83.8%) had heard about e-cigarettes. The prevalence of ever use of e-cigarettes (2.3%) [31] was similar to Taiwan (3%) [32] and China Mainland (3.1%) [33], but lower than many other countries (>6%) [34,35,36,37,38,39,40]. Previous studies suggested that the low prevalence may be due to the less aggressive e-cigarette promotion than western countries [31,41]. In contrast, local newspaper articles contained more reports on the potential harms of e-cigarettes and advocacy for e-cigarette regulation than those which showed the benefits of using e-cigarettes [42]. Therefore, the low prevalence of e-cigarette use and strong media advocacy on regulating e-cigarettes may underpin strong support for e-cigarette regulation. This study aimed to assess the public support for e-cigarette regulation, and factors associated with the support among a large sample of Hong Kong adults. Such findings will help understand the public opinion towards e-cigarettes and provide evidence for policy discussion and new legislation addressing e-cigarette use.

## 2. Materials and Methods 

### 2.1. Study Design and Data Collection

The Hong Kong Council on Smoking and Health (COSH, the Hong Kong statutory body for tobacco control advocacy) collaborated with the School of Public Health of the University of Hong Kong to conduct the Hong Kong Tobacco Control Policy-related Survey, and to gauge the public support for e-cigarette regulation. The present study was an analysis of the 2015 telephone-based survey, which used a two-stage random sampling, with over-sample of current and ex-smokers, and enquired opinions towards e-cigarette regulation among other questions. The survey was conducted by one of the most well-established survey agents in Hong Kong, i.e., the Public Opinion Programme, University of Hong Kong (HKU), from April to October 2015 on a random sample of Hong Kong residents aged 15 years or above. Telephone numbers were drawn randomly from residential telephone directories as seed numbers, from which another set of numbers were generated using the “plus/minus one/two” method so as to capture unlisted numbers. When telephone contact was successfully established with a target household, the family member whose birthday was the nearest to the interview date would be invited for the interview among all eligible family members at the time of the interview. Respondents completed a computer-based questionnaire administered by trained interviewers either in Cantonese or Mandarin, which are the mother tongues of 92% of the population [43]. Each interview took about 10–15 min to complete. All respondents gave informed consent before the survey started. The survey was approved by the Institutional Review Board of the University of Hong Kong/Hospital Authority Hong Kong West Cluster (Ref: UW15-108).

The respondents for this study included (a) current smokers who, at the time of the survey, used conventional cigarettes daily or consumed at least 1 cigarette in the past week (i.e., occasional use); (b) ex-smokers, who had used conventional cigarettes for at least 6 months in their lifetime but did not use at the time of the survey; and (c) never-smokers, who had never used conventional cigarettes in their lifetime. Smoking status was self-reported, which was deemed reliable in population-based surveys [44]. As the prevalence of current smokers (11.4% in 2015) and ex-smokers (6.2%) in Hong Kong was low [43], the recruitment for these smoking sub-groups was difficult. To stabilize the estimates for ex- and current smokers, these sub-groups were over-sampled. We targeted to recruit 1667 for each smoking group for the survey. The estimates of overall prevalence of support were then weighted by sex, age group, and smoking status of the general population from the 2015 Hong Kong census data. 

All respondents were asked the question: “Have you ever heard of electronic cigarettes? This is a device similar to cigarettes that can emit ‘vapor’ without combustion.” Only those who had heard or seen e-cigarettes were further invited to answer the questions on their use and opinions towards e-cigarettes, whereas the others were excluded from the analysis on perceived harmfulness of e-cigarettes and support for e-cigarette regulations.

### 2.2. Measurements

E-cigarette use was assessed by the question: “Have you ever used e-cigarettes?” Respondents answering “yes” were classified as ever users, and were then asked to report their frequency of use in the past 30 days. Respondents who had not heard of e-cigarettes were treated as non-e-cigarette users. 

Respondents’ support or not for e-cigarette regulation was assessed through 4 questions: “Do you agree the government should (1) ban the e-cigarette promotion and advertisements, (2) ban the use of e-cigarettes in smoke-free venues, (3) ban the sale of e-cigarettes to people aged under 18, and (4) restrict the sale of nicotine-free e-cigarettes such as by requiring registration before sale?” Response options were “Yes”, “No”, and “I don’t know”. 

Respondents’ perceived harmfulness of e-cigarettes was measured through the question: “Compared with conventional cigarettes, do you think e-cigarettes are more harmful or less harmful?” with response options of “e-cigarettes are much more harmful”, “e-cigarettes are a bit more harmful”, “both are similarly harmful”, “e-cigarettes are a bit less harmful”, “e-cigarettes are much less harmful” and “I don’t know”. These options were re-categorized as “e-cigarettes are more harmful”, “similar harmfulness”, “e-cigarettes are less harmful” and “I don’t know” in the analysis. Respondents’ sex, age, marital status, education level, and monthly household income were also enquired. 

### 2.3. Statistical Analysis

Estimation of overall prevalence and regression coefficients were adjusted using probability weights to match the distribution of sex, age and smoking status of the general population in Hong Kong. The corresponding estimates within each group of smoking status were weighted by sex and age distribution in each smoking status. The sex and age group distribution of Hong Kong’s mid-year general population in 2015 [45], and the prevalence of the 3 groups of smoking status from the Hong Kong Thematic Household Survey in 2015 [43], were used to construct the weight matrix. Cohen’s effect size (ω) was used to compare the distribution of sex, age, marital status and smoking status of our weighted sample to the general population. 

The overall distributions of awareness, use, perceived harmfulness of e-cigarettes, and support for the four e-cigarette regulations were analyzed by weighted descriptive statistics. Due to the high prevalence in supporting the regulations, we used four separate Poisson regression models (Stata command: glm and link(log)) with robust error variance (stata command: vce (robust)) to estimate prevalence ratios (PR, relative risk for outcome with high prevalence) for the association of the explanatory variables (smoking status, ever use of e-cigarettes and perceived harmfulness of e-cigarettes) and the public’s support for the four regulations, controlling for socio-demographic variables [46]. All data analyses were conducted using STATA (Version 13, TX: StataCorp LP, College Station, TX, USA).

## 3. Results

### 3.1. Sample Characteristics 

The survey agent generated and dialed 149,272 telephone numbers, in which 59,911 were non-eligible numbers; 83,290 numbers were valid telephone numbers but the eligibility could not be confirmed due to unanswered calls, incomplete screening, busy line, or no Chinese speakers; 23,497 numbers had an eligible person in the household for the interview, but the rescheduled survey could not be completed eventually within the study period. Among the 6071 eligible people contacted, 5252 (1706 current smokers, 1712 ex-smokers and 1834 never-smokers) provided oral consent and completed the interview, resulting in a response rate of 17.8%. Other eligible subjects were excluded, which were composed of 437 who did not complete the survey (7.2%), 183 refused to participate (3.0%), and 199 (3.3%) were not available at the survey time. Ex-smokers (survey sample 32.6% vs. HK population 6.2%), current smokers (32.5% vs. 11.4%), male (66.6% vs. 46.1%), aged 60 years or above (38.2% vs. 24.7%), married male (72.3% vs. 61.4%) and married female (60.4% vs. 55.4%) were over-sampled in the unweighted sample. Upon weighting with the census population statistics, the distribution of all socio-demographic characteristics was similar to the general population (all Cohen’s **ω** < 0.3) (Table 1). Education level varied by smoking status, such that more ex-smokers (20.8%) than never (11.7%) and current smokers (12.8%) attained primary education, and more never smokers (41.9%) than ex- (25.4%) and current smokers (26.5%) attained tertiary education. 

### 3.2. Descriptive Statistics of Key Variables 

In all respondents regardless of smoking status, over 80% had heard of e-cigarettes (Table 2). Only 0.7% of all respondents had ever used e-cigarettes and 0.2% reported current e-cigarette use in the past 30 days. More current smokers reported ever and current use than never- and ex-smokers. 

In all respondents who had heard of e-cigarettes, about one-third (32.9%) thought that e-cigarettes are less harmful than conventional cigarettes, one-fifth (21.8%) thought they are similarly harmful, and one-fourth (27.8%) did not know the harmfulness (Table 3). Fewer current smokers (21.6%) than ex-smokers (24.3%) and never-smokers (34.7%) perceived that e-cigarettes are less harmful than conventional cigarettes. 

In the respondents who had heard of e-cigarettes, their support for banning e-cigarette promotion and ads (71.7%) was lower than that for banning e-cigarette sale to people aged under 18 (93.9%), banning e-cigarette use in smoke-free areas (81.5%), and regulating the sale of nicotine-free e-cigarettes (80.9%). Only about 2.8% of all the respondents did not support any e-cigarette regulation, and the corresponding proportion for never, ex- and current smokers were 2.6%, 2.5% and 4.2%, respectively. In contrast, over half (57.8%) supported all four regulations. Current smokers showed a lower support for all four regulations than never- and ex-smokers.

### 3.3. Correlates of Support for E-Cigarette Regulation

Controlling for socio-demographic characteristics, being a current smoker was associated with lower support for the four e-cigarette regulations (all PR < 1, *p* < 0.01, reference group: never-smokers) (Table 4), whereas the support in never- and ex-smokers was similar. Ever users of e-cigarettes had lower support for the four regulations than never-users, but the PRs were insignificant due to the very small number of e-cigarette users. Perceiving “e-cigarettes and conventional cigarettes are similarly harmful” and “e-cigarettes are more harmful” were associated with more support for the four e-cigarette regulations (all PR > 1, *p* < 0.01), compared with those who responded “don’t know” towards e-cigarette harmfulness. Perceiving “e-cigarettes as less harmful than conventional cigarettes” and not knowing the harmfulness showed a similar level of support in the regulations, except for restricting the sale of nicotine-free e-cigarettes (79.1% vs. 72.5%, PR = 1.19, 95% CI 1.12–1.27).

## 4. Discussion

This is the first study to examine the public support for e-cigarette regulation in a large sample of Chinese residents. The awareness of e-cigarettes is high but the prevalence of e-cigarette use is low in Hong Kong. A great majority (72–94%) of respondents supported the four e-cigarette regulations, including banning e-cigarette promotion and ads, e-cigarette use in smoke-free venues, e-cigarette sale to minors, and restricting sale of nicotine-free e-cigarettes. Being a current smoker, and perceiving e-cigarettes as less harmful or not knowing the harmfulness was associated with a lower level of support. 

The strong support for the e-cigarette regulation was consistent with the recent local content analysis showing that local newspaper articles on e-cigarettes mostly reported the harmful effects of e-cigarettes [42], which might have increased the perceived harmfulness in the public. Moreover, the current vigorous regulation of e-cigarettes probably led to the lower exposure and acceptability of the products. For example, the licensing for nicotine products have greatly reduced the availability and marketing of e-cigarettes as cessation aids. The ban on vaping in smokefree venues may have also limited the public exposure of the products. Lastly, the widespread anti-smoking culture and the comprehensive tobacco control measures in Hong Kong, including the restriction of all tobacco advertisements, and the smoking ban in most public areas, may have de-normalized smoking and contributed to strong public support for regulating the new emerging tobacco products. 

Our study showed a strong public support for restricting the sale of nicotine-free e-cigarettes. Sale of nicotine-free e-cigarettes is not restricted according to the current legislation, which implicitly positions them as consumer goods that could be purchased by all people. However, our finding showed that the general public did not agree to this grey area of policy, and supported the government to restrict the sale of these products. Although the e-cigarette advertising claims that the e-liquid had no nicotine, their potential health hazards due to chemical substances and flavouring agents should not be neglected. The effectiveness of nicotine-free e-cigarettes has not been tested, so that using them as smoking cessation tools should not be encouraged at this stage. Therefore, promoting the use of these products for leisure purpose should be regulated. As legislation and the implementation of new regulations on e-cigarettes could take a longer time, more monitoring on the marketing of nicotine-free e-cigarettes are needed. 

The banning of e-cigarette promotion received less public support than the other three e-cigarette regulations. Similar difference was observed during the early stage of tobacco control advocacy in the 1990s, when the Hong Kong government proposed to ban tobacco advertisements and restrict smoking in indoor public areas. Similar random household telephone surveys at that time showed that the public support for a total ban of tobacco advertisements (50%) was lower than that for a total ban of cigarette-smoking in shops and restaurants (77%) [47,48]. These findings further showed that the general public may be less resistant to tobacco promotions and ads. To increase public support for banning e-cigarette promotion and advertisement, the public should be well informed about the adverse impacts of e-cigarette marketing. For instance, evidence on the surging youth exposure to e-cigarette promotions [49], and the increase in youth susceptibility and prevalence of e-cigarette use due to these promotions [50,51,52] should be considered and publicized.

We found that some people were not clear about the harmfulness of e-cigarettes, despite the high awareness. The possible reasons of choosing “don’t know” when asked about e-cigarette harmfulness were the exposure to conflicting information on e-cigarettes from the media and smoking peers [23], and their uncertainty about the long-term safety and effectiveness of e-cigarettes. As shown in the regression model, these people showed a lower support for e-cigarette regulation. To further increase public support for e-cigarette regulation, more evidence on the safety and long-term health effects of e-cigarette use, and effective health communication of these evidences is needed.

### Limitations

Our study had some limitations. Firstly, about 14% of respondents had not heard of e-cigarettes and hence were not asked about their perception on e-cigarette harmfulness and their support towards regulation. Secondly, the causal relationship between the variables is uncertain in this cross-sectional study. Longitudinal study is a better way to assess causal associations. Thirdly, due to the limited time of each telephone interview (10–15 min) and hence limited number of questions, our study did not include other regulatory measures like the requirement to display health claims and health warning labels on e-cigarette packaging. Our questions of the three regulatory measures did not distinguish between e-cigarettes containing nicotine or not, hence the findings reflected the respondents’ view on e-cigarettes in general. Fourthly, the low response rate (17.8%) may reduce the representativeness of the sample. Lastly, the respondents might provide socially desirable answers to support e-cigarette regulation, but the impact should be minimal because the telephone survey was voluntary and anonymous. 

## 5. Conclusions

The general public in Hong Kong favors e-cigarette regulation, which supports the Hong Kong government proceeding with the legislation. Current stringent measures on tobacco and e-cigarettes, and media reports on the harmfulness of e-cigarettes may underpin the strong support for the regulations. Current smokers, and people who perceived e-cigarettes as less harmful or did not know the harmfulness of e-cigarettes had lower support for the regulation. 

## Figures and Tables

**Table 1:** Unweighted and weighted figures of baseline socio-demographic characteristics (*n* = 5252).

		Survey Sample				Cohen’s ω	
		Unweighted%	(1) Weighted% 1	(2) Weighted% 1,2	(3) HK Population% 3	(1) vs. (2)	(1) vs. (3)
		n = 5252	n = 5252	n = 4517			
**Smoking**	Never-smoker	34.9	84.3	83.1	82.5	0.05	0.03
**Status**	Ex-smoker	32.6	5.5	5.6	6.2		
	Current smoker	32.5	10.2	11.3	11.4		
**Sex**	Male	66.6	45.4	48.5	46.1	0.01	0.05
	Female	33.4	54.6	51.5	53.9		
**Age group**	15–29	14.4	20.1	21.8	20.1	0.00	0.07
	30–39	10.1	17.7	17.3	17.7		
	40–49	16.0	17.9	18.4	17.9		
	50–59	21.2	19.6	20.5	19.6		
	60 or above	38.2	24.7	22.0	24.7		
**Education Level**	Primary and below	17.4	12.4	10.3	18.9	0.22	0.29
	Secondary	53.4	48.3	47.3	50.3		
	Post-secondary	29.2	39.3	42.4	30.8		
**Marital status**	Single	18.9	34.4	34.9	33.5	0.07	0.07
**(Male)**	Married	72.3	62.0	61.5	61.4		
	Divorced/widowed	8.8	3.6	3.6	5.1		
**Marital status**	Single	27.4	31.3	33.1	29	0.26	0.28
**(Female)**	Married	60.4	62.5	61.5	55.4		
	Divorced/widowed	11.8	6.2	5.4	15.6		
**Monthly household**	<10,000	18.8	12.7	10.8	-	Not available	
	10,000–19,999	14.5	13.3	13.2	-		
**Income**	20,000–29,999	14.0	13.5	13.5	-		
**(HK$, US$1 = HK$7.8)**	30,000–39,999	11.8	12.4	2.6	-		
	≥40,000	22.3	26.1	28.6	-		
	Missing	18.6	22.0	21.4	-		

^1^ All descriptive values are percentages weighted by age and sex distribution of the Hong Kong population (2015), and smoking prevalence in the Hong Kong Thematic Household Survey (2015, Report No. 59 [43]); ^2^ Respondents who had not heard of e-cigarettes (*n* = 735) were excluded; ^3^ Data source from Hong Kong Monthly Digest of Statistis, June 2016 and Women and Men in Hong Kong, July 2015, published by Census & Statistics Department, Hong Kong Special Administrative Region Government.

**Table 2:** Prevalence of being aware of e-cigarettes, and use of e-cigarettes in all respondents, weighted % (95% Confidence Interval (CI)).

	Never-Smokers	Ex-Smokers	Current Smokers	Total
	*n* = 1834	*n* = 1712	*n* = 1706	*n* = 5252
Had heard of e-cigarettes	82.6 (80.8, 84.3)	86.4 (84.7, 88.0)	92.4 (91.0, 93.5)	83.8 (82.2, 85.3)
Ever use of e-cigarettes	0.1 (0.04, 0.4)	0.6 (0.3, 1.1)	5.5 (4.4, 6.9)	0.7 (0.5, 0.9)
Used e-cigarettes in past 30 days	0.0	0.09 (0.02, 0.4)	1.7 (1.1, 2.6)	0.2 (0.1, 0.3)

**Table 3:** Perception towards e-cigarettes, and support for e-cigarette regulation, by smoking status in respondents who had heard of e-cigarettes, weighted % (95% CI).

	Never-Smokers	Ex-Smokers	Current Smokers	Total
	*n* = 1510	*n* = 1470	*n* = 1537	*n* = 4517
Perceived harmfulness (compared with conventional cigarettes)				
E-cigarettes are more harmful	16.5 (14.7, 18.6)	17.5 (15.6, 19.5)	25.2 (22.9, 27.7)	17.6 (16.0, 19.3)
E-cigarettes and cigarettes are similarly harmful	22.0 (19.9, 24.2)	20.8 (18.8, 23.1)	20.6 (18.5, 22.9)	21.8 (20.0, 23.7)
E-cigarettes are less harmful	34.7 (32.3, 37.3)	24.3 (22.1, 26.6)	21.6 (19.4, 24.0)	32.9 (30.8, 35.0)
Don’t know	26.8 (24.5, 29.2)	37.4 (34.9, 40.0)	32.6 (30.2, 35.1)	27.8 (25.9, 29.8)
Total	100.0	100.0	100.0	100.0
Support for e-cigarettes regulation (prevalence by smoking status)				
Ban e-cigarette promotion and ads	73.4 (71.0, 75.7)	74.2 (71.8, 76.4)	57.7 (55.1, 60.4)	71.7 (69.7, 73.6)
Ban e-cigarette use in smoke-free venues	82.9 (80.8, 84.8)	79.9 (77.7, 81.9)	71.4 (68.9, 73.8)	81.5 (79.8, 83.1)
Ban sale to people aged under 18	94.3 (92.9, 95.4)	93.7 (92.3, 94.9)	90.5 (88.8, 91.9)	93.9 (92.7, 94.8)
Restrict sale of nicotine-free e-cigarettes	82.0 (80.0, 84.0)	79.2 (77.1, 81.3)	73.3 (70.9, 75.6)	80.9 (79.2, 82.5)
Number of supported regulations (per person)				
0	2.6 (1.9, 3.6)	2.5 (1.8, 3.4)	4.2 (3.3, 5.3)	2.8 (2.2, 3.6)
1	3.7 (2.8, 4.8)	5.8 (4.7, 7.1)	8.9 (7.5, 10.6)	4.4 (3.6, 5.3)
2	12.1 (10.5, 13.9)	11.1 (9.6, 12.9)	18.4 (16.4, 20.6)	12.7 (11.3, 14.2)
3	21.5 (19.5, 23.8)	23.5 (21.4, 25.8)	26.9 (24.6, 29.4)	22.3 (20.5, 24.2)
4	60.0 (57.4, 62.5)	57.1 (54.5, 59.7)	41.6 (39.0, 44.3)	57.8 (55.6, 60.0)
Total	100.0	100.0	100.0	100.0

**Table 4:** Support for e-cigarette regulations by smoking status, ever use of e-cigarettes and perceived harmfulness of e-cigarettes in the respondents who had heard of e-cigarettes.

	Ban Promotion and Ads			Ban Use in Smoke-Free Venues			Ban Sale to Minors			Restrict Sale of Nicotine-Free E-Cigarettes		
	%	Adj. PR	*p*-Value	%	Adj. PR	*p*-Value	%	Adj. PR	*p*-Value	%	Adj. PR	*p*-Value
Smoking status												
Never-smoker (*n* = 1510)	73.4	1		82.9			94.3	1		82.0	1	
Ex-smoker (*n* = 1470)	74.2	0.98 (0.93–1.04)	0.49	79.9	0.97 (0.93–1.01)	0.17	93.7	1.00 (0.97–1.02)	0.69	79.2	0.97 (0.93–1.01)	0.17
Current smoker (*n* = 1537)	57.7	0.77 (0.72–0.81)	<0.01	71.4	0.86 (0.82–0.90)	<0.01	90.5	0.96 (0.93–0.98)	<0.01	73.3	0.88 (0.84–0.92)	<0.01
Ever use of e-cigarettes												
No (*n* = 4428)	71.9	1		81.7	1		93.9	1		81.0	1	
Yes (*n* = 89)	47.2	0.79 (0.61–1.03)	0.08	63.0	0.84 (0.67–1.06)	0.15	85.3	0.93 (0.81–1.05)	0.25	68.9	0.92 (0.77–1.11)	0.38
Perceived harmfulness (compared with conventional cigarettes)												
E-cigarettes are more harmful (*n* = 864)	88.3	1.41 (1.31–1.52)	<0.01	89.4	1.20 (1.13–1.26)	<0.01	96.7	1.07 (1.04–1.10)	<0.01	86.3	1.19 (1.12–1.27)	<0.01
Similar harmfulness (*n* = 924)	84.7	1.34 (1.25–1.45)	<0.01	90.1	1.18 (1.12–1.25)	<0.01	97.2	1.08 (1.04–1.11)	<0.01	90.0	1.23 (1.16–1.31)	<0.01
E-cigarettes are less harmful (*n* = 1194)	61.3	0.96 (0.88–1.06)	0.44	76.9	1.00 (0.94–1.07)	0.94	93.0	1.03 (0.99–1.06)	0.15	79.1	1.19 (1.12–1.27)	0.03
Don’t know (*n* = 1535)	71.7	1		71.5	1		90.4	1		72.5	1	

PR = prevalence ratio. All the four regression models were adjusted for sex, age group, marital status, education level, and monthly household income.
